# The Story of the Hospitals

**Published:** 1887-03-12

**Authors:** 


### The Story of the Hospitals.

(*Continued from p.* 328.)

### The Central London Throat and Ear Hospital.

Special Hospitals.

The close connection which exists between the throat and ear, and consequently between the diseases to which these two organs are subject, affords an ample *raison d'être* for such institutions as the above. Doctors have quarrelled, and to a certain extent still quarrel, over the value of special hospitals; and doubtless their indiscriminate multiplication for the treatment of disease of every organ of the human frame would be fraught with evil. Within certain limitations, however, practical experience has made out a very good case in their favour. Diseases of the eye, the teeth, the ear and throat, the skin, physical malformation, etc., are undoubtedly better dealt with in special hospitals, or at any rate in special departments and by special persons, and it is the recognition of this fact which has led to the establishment of "special departments" at the great general hospitals. The ills to which flesh is heir range over far too wide a field for their exhaustive study and treatment to come within the compass of a single life. If a watch is the better made when its several parts are prepared by different hands, it is easy to understand that the human body—a much more complex piece of mechanism—is more skilfully dealt with when its leading diseases become the objects of special study.

Foundation of the Central.

The Central London Throat and Ear Hospital is situated in the Gray's Inn Road. Externally it is an unpretentious institution, with little to attract attention save its name, and the inscription: "Entirely Free to the Indigent Poor." Its founder was Mr. Lennox Browne, F.R.C.S., its senior surgeon, assisted by Dr. Llewelyn Thomas (also one of its surgeons from the first) and a few private friends, including Captain Hutton, Mr. Ernest Turner, and Mr. George Wallis. The original idea arose from the fact that, at the time of its foundation, there was but one hospital in the metropolis—that in Golden Square — which was really devoted to diseases of the throat. At the London general hospitals, at that time, also, there were few that had ear departments, and not more than one or two that specially treated the throat. Moreover, where a throat department was to be found, the surgeon in attendance was mostly not a specialist, but one of the general assistant surgeons told off for the work. The birth of the Central London Throat and Ear Hospital took place on March 25, 1874. Its very early infancy was passed in humble, rented premises in Manchester Street, off the Gray's Inn Road. The work of the first twelve months convinced the committee very unmistakably of the extreme need which existed for a central institution, conducted on the principle of a hospital for the treatment of diseases of the throat, ear, and related organs. During this first year, the little hospital gave relief to no less than 3,018 patients; 1,202 being throat cases, 811 ear cases, and 1,005 throat and ear disease combined. All this was done with a modest income, amounting to only a trifle over a thousand pounds.

The Present Premises.

The foundation-stone of the present premises of the hospital was, appropriately enough, laid by that queen of song, Madame Adelina Patti, September 16, 1875; the work of erection went on with all speed, and, within a twelvemonth, the new hospital was open and in full working order. I need not here pause to describe in detail the work of the next ten years. It has shown an incessant increase, a fact which sufficiently testifies to its growing repute, and to the excellence of its management. From the Twelfth Annual Report I gather that, during the twelve months ending 25th March last, the new out-patients numbered 4,946, the attendances made amounting to 24,447, while 237 persons were received as in-patients, while the available income stood at £1,700.

Evening Attendance.

The committee from the first saw the importance of evening attendances. The bread-winner, engaged during the day, is too often unable to get away from his work, and cannot loiter in out-patient waiting-halls "for the doctor." So, not improbably, a disease which might be easily grappled with in its incipient form, is left to take root, and "the doctor" is consulted only when it is too late, or, at least, when the case is far more difficult to cure than it would have been if early application had been made. Advantage has been so generally taken of the evening attendance, that the committee were, not very long since, compelled to change the hour from six to five, in order to deal with the increasing pressure of cases.

Out-Patient Contributions.

I notice in the hospital balance sheet for the year to which I am referring, a sum of £717 0*s.* 6*d.* received as contributions from out-patients. Bearing in mind the modest income of the hospital, this is an item of no small importance. It is a well-known fact that the great majority of persons who frequent the waiting-halls of our hospitals can afford to pay something, and this being so, there can be no reason why they should not be asked to contribute. Philanthropy is not an accident more among doctors than among other individuals. They do not fight for hospital appointments solely for the sake of doing good to their fellow-creatures. The experience which they gain from out-patient practice may perhaps be regarded in a measure as an equivalent for the advice they give. But advice must, in most cases, be accompanied by medicine, and in the case of in-patients, by board. If there is a set-off against the skill which the doctor is enabled to acquire, there is no reason why the patient should receive *gratis* his medicine and food, which costs money, unless it is a case of unquestioned poverty. The out-patient system at the Central Throat is based on this line of reasoning. The applicant, on registering his name, etc., is asked as to his occupation and earnings. Then comes the question, "Can you afford to pay a little contribution?" in most cases practically answered by sixpence or a shilling being put down. An experienced questioner—and I was surprised to see the secretary's cleverness in this direction—is able to take pretty accurate "stock" of his applicant, and a skilfully applied interrogatory will generally dispose of the case. I need scarcely say that poor patients who are passed free receive equal attention with others, *no distinction whatever being made.*

Inside the Hospital.

In company with the Secretary, I made a tour of the wards of this institution. Each I found the picture of good order, neatness, and cleanliness. They have been named after the benefactors of the institution. In the Llewelyn Thomas Ward is an interesting tablet to the memory of this good man, whose cherished name in connection with the Central Throat will die only with the institution itself. The tablet reads: "In memory of Llewelyn Thomas, M.D., one of the founders of the hospital, and an unwearied member of the medical staff up to the day of his death, November 26, 1884." Among the other wards are the Canterbury, in compliment to their archiepiscopal President; the Lofty, named after Miss Lofty, one of the hospital's earliest and most devoted friends; Adelina Patti, and the Lennox Browne. Mention must also be made of Miss Dora Jackson, another excellent friend of the hospital, who, beyond collecting from all her friends with perennial increase of success, has had the happy thought of sending separate pretty bouquets for each in-patient during the summer months. The blue and white glazed tiles around the lower portion of the hospital, along the staircases, waiting-room, and consulting and operating chambers, give a very clean and pleasant effect, besides being a vast improvement upon colour-washed walls. The expense of these tiles is, I presume, the reason why they are not more generally employed in hospitals; in the case of the institution I am describing, I am informed that Messrs. Trollope were kind enough to supply them at cost price. In any case, the interest on first cost would not cover half the expense of a yearly cleaning and one coat of paint. Rather a novel feature has been planned on the ground-floor. The out-patient, after registering his name, etc., passes to the left to the waiting-halls, and from these are reached the consulting, examining, and operating rooms and dispensary, the patient quitting the hospital on the side opposite to that on which he entered, having made, as it were, a complete circuit.

A Novel Case.

The surgeon in attendance, in the course of a most interesting conversation which I had with him, related to me some of the more exceptional cases brought to this hospital. I had explained to me the marvellous searchings which the laryngoscope can make of the human throat, its revelations of the voice-box, and how it lays bare disease at its very threshold. The connection of the throat with the ear and the passages of the nose were also demonstrated. I was told of one case of "loss of voice" which was passing strange. The "sufferer" was a young man, a sergeant in a Guards regiment. As there seemed to be no possible reason for the loss of voice, nor why so promising a young officer should "malinger," he was brought to the hospital. But the patient had overdone his malady, and by simulating loss of *speech* as well as of *voice*, at once showed the case to be one of imposition. Galvanism was used with the most efficacious result of restoring both voice and speech, and I am sorry to say the interesting young soldier was an impostor!

In the Wards.

It must not be supposed, however, that all the cases are of such a simple though sensational character. In the wards I saw patients suffering from consumption affecting the throat, the most painful manifestations of this distressing malady. I was informed that there is great promise that much of this suffering will be relieved, and indeed that in some cases may even be cured, by recent improvements in treatment. One poor girl, only twenty-two, was the victim of cancer, and for nearly two months had taken but a few drops of fluid by the mouth a day. Cases of ear disease extending to the brain are also among the more serious in the aural department; while as to the nose, I was quite astonished to see how many affections there can be of this prominent but variable feature of the face. One otherwise very pretty girl I saw wearing an instrument—the invention of one of the surgeons—for correcting deflections of the dividing septum of the nostril, rendering the nose quite crooked.

### AMPLE COMPENSATION.

The late eminent French surgeon, Nélaton, had some fine shooting, to which he used to invite his friends. One day a careless sportsman had the misfortune to lodge some shot in the legs of a peasant, who crossed his fire just as he was discharging his gun at a hare. Piteous were the fellow's cries, as he pointed out his wounds to the illustrious surgeon; and, while the shot were being extracted, he indulged in some rather strong language at the expense of the aggressor. "What do you mean?" at last cried the exasperated sportsman. "You have the conscience to complain when I have procured you the honour of being attended by Nélaton for—nothing!"

